# Correlating Grain Boundary Character and Composition in 3‐Dimensions Using 4D‐Scanning Precession Electron Diffraction and Atom Probe Tomography

**DOI:** 10.1002/smtd.202401650

**Published:** 2025-02-28

**Authors:** Saurabh M. Das, Patrick Harrison, Srikakulapu Kiranbabu, Xuyang Zhou, Wolfgang Ludwig, Edgar F. Rauch, Michael Herbig, Christian H. Liebscher

**Affiliations:** ^1^ Max‐Planck‐Institut for Sustainable Materials (Max‐Planck‐Institut für Eisenforschung) Max‐Planck‐Straβe 1 40237 Düsseldorf Germany; ^2^ SIMAP Laboratory CNRS‐Grenoble INP BP 46 101 rue de la Physique Saint Martin d'Hères 38402 France; ^3^ ESRF–The European Synchrotron 71 Av. des Martyrs Grenoble 38000 France; ^4^ MATEIS INSA Lyon UMR 5510 CNRS 25 av Jean Capelle Villeurbanne 69621 France; ^5^ Research Center Future Energy Materials and Systems Ruhr Univeristy Bochum Universitätsstr. 150 44801 Bochum Germany; ^6^ Faculty of Physics and Astronomy Ruhr Univeristy Bochum Universitätsstr. 150 44801 Bochum Germany

**Keywords:** 4D scanning precession electron diffraction tomography (4D‐SPEDT), atom probe tomography (APT), correlative 3D‐TEM/APT, grain boundary character, nanocrystalline materials, segregation

## Abstract

Grain boundaries (GBs) are dominant imperfections in nanocrystalline materials that form a complex 3D network. Solute segregation to GBs is strongly coupled to the GB character, which governs the stability and macroscopic properties of nanostructured materials. Here, a 3D transmission electron microscopy and atom probe tomography (APT) correlation framework are developed to retrieve the GB character and composition at the highest spatial resolution and chemical sensitivity by correlating 4D scanning precession electron diffraction tomography (4D‐SPEDT) and APT on the same sample. The 3D GB habit plane network and explore the preferential segregation of Cu and Si in a nanocrystalline Ni‐W alloy is obtained. The correlation of structural and compositional information reveals that Cu segregates predominantly along high‐angle GBs and incoherent twin boundaries, whereas Si segregation to low‐angle and incommensurate GBs is observed. The novel full 3D correlative approach employed in this work opens up new possibilities to explore the 3D crystallographic and compositional nature of nanomaterials. This lays the foundation for both probing the true 3D structure‐chemistry at the sub‐nanometer scale and, consequentially, tailoring the macroscopic properties of advanced nanomaterials.

## Graphical abstract

A 3D correlative approach combining 4D scanning precession electron diffraction tomography and atom probe tomography on the same specimen unlocks the structure and chemistry of grain boundary networks in nanocrystalline materials. This approach enables to determination of local crystallographic parameters for any nonplanar general type of interface together with local elemental segregation at nanometer resolution.

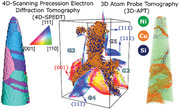

## 1 Introduction

The structure and composition of interfaces such as phase boundaries, grain boundaries (GBs), and twin boundaries play a vital role in controlling the macroscopic properties of polycrystalline materials. Moreover, the behavior of materials such as thermal stability, fracture toughness, electrochemical properties, hydrogen embrittlement, and electrical conductivity can be tailored through the design of interface structure and composition.^[^ ^1^, ^2^, ^3^ ^]^ Generally, the structure of any interface that separates differently oriented crystals, termed GB, can be described by five independent crystallographic parameters (macroscopic degrees of freedom, DOF). Three of them describe the mutual misorientation of the adjoining crystals and two define the inclination of the interface (interface habit plane). For the case of a coherent twin boundary in the face‐centered cubic (fcc) system with undisrupted atomic periodicity, both crystals are symmetrically rotated by 60° around a common [110] misorientation axis. The corresponding GB habit plane $\left(1\bar{1}1\right)$ is perpendicular to this tilt axis and defines the plane of the interface. Additional GB defects have to be incorporated at the interface to compensate for the deviation from the $\left(1\bar{1}1\right)$ twining plane, leading to the formation of incoherent twin segments with typically $\left(1\bar{1}2\right)$ habit plane^[^ ^4^ ^]^ The enrichment of solute elements at these interfaces, termed segregation, has been shown to strongly depend on the GB parameters and adds another level of complexity to the determination of interfacial, and hence material properties^[^ ^1^ ^]^ GBs typically span a complex 3D network within polycrystalline materials with locally varying interface parameters confined to within several nanometers. As a result, due to the locality and scale of these interfaces, the development of novel characterization techniques is required to retrieve their structural and compositional information in 3D and at the highest possible spatial and compositional resolution.

Structural information on crystal orientations and interfaces is typically resolved down to atomic resolution by Scanning and Transmission Electron Microscopy (SEM and TEM). Electron Backscatter Diffraction (EBSD) in the SEM and Scanning Precession Electron Diffraction (SPED) in the TEM are widely used to reveal crystal misorientations and geometric parameters of GBs from single projections.^[^ ^5^, ^6^ ^]^ Atomic resolution scanning transmission electron microscopy is capable of resolving the atomic nature of GBs, however, mostly limited to special GBs with a common tilt or twist axis.^[^ ^7^, ^8^ ^]^ 3D techniques, such as 3D X‐ray Diffraction Microscopy or serial sectioning EBSD, have been established to determine the complete crystallography of polycrystalline materials and the GBs therein with millimeter down to sub‐micrometer resolution.^[^ ^9^, ^10^, ^11^, ^12^ ^]^ It has recently been demonstrated that the 3D atomic structure of GBs can be resolved by atomic electron tomography with sub‐Angstrom resolution in a relatively small field of view of several tens of square nanometers^[^ ^13^ ^]^ Over the past decade, two TEM‐based orientation mapping techniques, dark‐field conical scanning, and SPED, have been coupled with tomography tilt series acquisition to obtain 3D crystal orientations with nanometer resolution.^[^ ^14^, ^15^, ^16^, ^17^ ^]^ While these techniques provide high‐resolution and 3D structural information, the associated local 3D distribution of elements at the interfaces remains elusive.

Spectroscopic methods in electron microscopy, such as Energy Dispersive X‐ray (EDX) or Electron Energy Loss Spectroscopy (EELS), probe compositional information with high spatial resolution from 2D projections of a sample, however, are limited by their ability to detect low elemental concentrations or light elements^[^ ^18^ ^]^ Atom Probe Tomography (APT), on the other hand, can obtain 3D elemental distributions of materials with both high spatial resolution, down to the sub‐nanometer level, and high chemical sensitivity, at the parts‐per‐million (ppm) level^[^ ^19^ ^]^ Microstructural features such as dislocations, stacking faults, and GBs can only be indirectly revealed if solute elements are segregating or depleting at these material imperfections.^[^ ^20^, ^21^ ^]^ Crystallographic information can also be obtained by APT, however, in very limited scenarios and usually restricted to grain orientation analysis.^[^ ^22^, ^23^ ^]^ To determine both crystallographic and compositional information with the highest possible resolution from the same sample location, techniques correlating electron microscopy with APT have been developed. Seminal correlative approaches were performed in the 1960 s by including target preparation of GB regions.^[^ ^24^, ^25^, ^26^ ^]^ Refined sample preparation strategies have enabled a direct correlation of TEM and APT on the same sample.^[^ ^27^, ^28^, ^29^ ^]^ These correlative approaches have provided novel insights into a wide variety of material science‐related phenomena, more specifically grain boundary segregation engineering (GBSE).^[^ ^30^, ^31^, ^32^ ^]^ Herbig et. al. have demonstrated that by combining scanning nanobeam diffraction mapping and APT, the local 2D GB crystallography and 3D elemental distribution can be correlated in a nanocrystalline sample^[^ ^30^ ^]^ However, these correlative TEM/APT techniques can only fully characterize the 5‐DOFs for planar interfaces parallel to the electron beam direction as exemplified for columnar nanocrystalline pearlitic steel or line segregation at faceted Si GBs.^[^ ^4^, ^30^ ^]^ The vast majority of nanocrystalline materials exhibit more complex 3D grain shapes and boundary networks, which could not be accessed by correlative techniques, so far.

Direct correlation of 3D crystal orientation mapping in the TEM and APT can mitigate the limitations of the individual techniques and enable a holistic characterization of complex nanomaterials in 3D with nanometer resolution^[^ ^33^ ^]^ Establishing relationships between local crystallography and composition in 3D can also be applied to observe reaction mechanisms in nanostructured electrocatalysts, the phase evolution of crystalline nanoprecipitates in nanostructured soft‐magnetic alloys, the development of oxide‐alloy interfaces during materials degradation, and advanced lithium‐ion battery cathodes.^[^ ^34^, ^35^, ^36^, ^37^, ^38^ ^]^ Obtaining both crystallographic and compositional information in all 3D lays the foundation for understanding material behavior and evolution down to the atomic level. Here, we develop a full 3D correlative approach combining 4D‐scanning precession electron diffraction tomography (4D‐SPEDT) with APT on the same sample. This technique enables to obtain 3D crystal shapes and orientations and a complete 5‐parameter description of local GB properties, as well as their associated 3D segregation levels in nanomaterials. We apply this method to nanocrystalline Ni–W alloys electrodeposited on Cu substrate due to their exceptional coarsening resistance and propensity to form equiaxed nano‐grains. Our 3D correlative approach enables systematic analysis of the interrelation of local GB crystallography and elemental segregation of Cu and Si with unprecedented resolution, thus paving the way toward integrated structure‐composition characterization of nanomaterials.

## 2 Result

**Figure 1:**
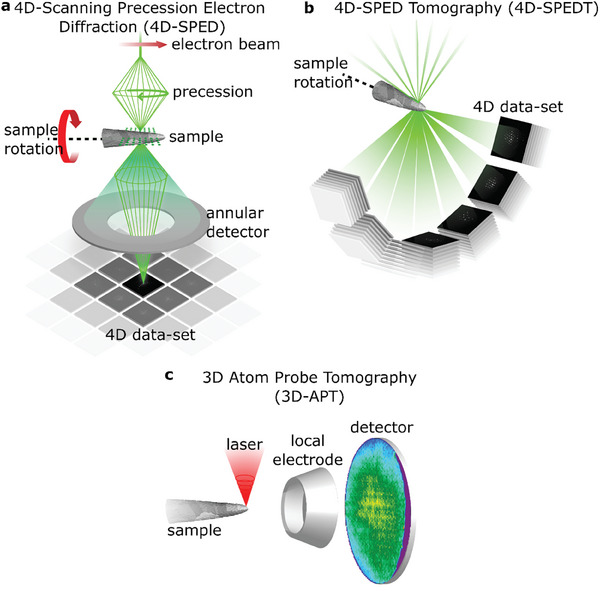
Schematic illustration of the data acquisition protocol. a) 4D‐SPED technique where a precessed nano‐sized beam is scanned over a needle‐shaped sample and electron diffraction patterns are recorded pixel by pixel using a pixelated detector. b) The 4D‐SPED dataset is acquired tilt by tilt in the range from −80° to +80° at a tilt step of 10° using an on‐axis rotation tomographic holder. The sample is cleaned in a low‐kV Argon ion shower before being loaded into the APT chamber. c) The sample is field evaporated using electric and laser pulses and atoms are collected on a position sensitive detector.

To link GB character and segregation with highest possible spatial and chemical resolution in 3D, we correlate 4D‐SPEDT and 3D‐APT on the same sample. The three main steps of the workflow are shown in **Figure** **1**: 1) Acquisition of a 4D‐SPEDT (see Figure 1a,b) series for grain orientation and GB reconstruction, 2) 3D‐APT (Figure 1c) to obtain compositional information from the same sample, and 3) data processing to combine the grain orientation and undary crystallography from 4D‐SPEDT with the elemental distribution from APT.

### 2.1 Data Acquisition for 3D Structure‐Composition Correlation

To determine the 3D crystallographic information (e.g., 5 DOFs of the GBs), 4D‐SPED was performed at each tilt angle throughout the tilt series. In this technique (Figure 1a), a nano‐sized incident beam is processed by 1° whilst being scanned over a needle‐shaped sample of a Ni‐W nanocrystalline alloy to realize quasi‐kinematical scattering conditions. The diffraction patterns are recorded at each probe position using a scintillator‐coupled complementary metal–oxide–semiconductor (CMOS) camera. The resulting 4D dataset contains spatially resolved crystallographic information. A 4D dataset was recorded at each 10° tilt increment over a ±80° tilt range, as illustrated in Figure 1b. The same needle‐shaped sample was subsequently measured by APT to obtain the 3D distribution of elements in the sample as illustrated in Figure 1c.

### 2.2 3D Crystal Orientation Mapping

**Figure 2:**
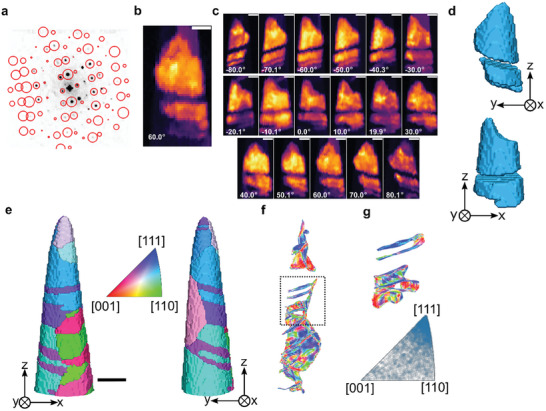
a) ACOM illustration demonstrating the correlation between the best‐matched diffraction template (open red circle) and an experimental diffraction pattern extracted from 60° tilt dataset. b) The orientation‐specific virtual dark field image calculated using these virtual apertures (open red circle). c) Orientation‐specific virtual dark field images of a grain tracked through the entire tilt series. d) The 3D reconstructed isosurface rendering of this grain in two different view angles. e) The 3D reconstructed isosurface rendering of the Ni‐W nanocrystalline alloy in two different view angles. Each grain is colored by its average orientation projected along the *z*‐axis. f) GB habit planes were reconstructed from the 3D reconstructed volume shown in (e) and segmented into triangular mesh. Mesh faces are colored according to their crystallographic plane orientation in inverse pole figure (IPF) color. g) The GBs from the highlighted (black dashed line) region in (f) and its GB habit plane normal distribution plot. Scale bars are 50 nm.

Automated crystal orientation mapping (ACOM) was used to obtain the crystal orientation on a pixel‐by‐pixel basis for each tilt dataset by comparing the experimental nanobeam diffraction patterns to a simulated template library^[^ ^33^ ^]^ **Figure** **2**a illustrates the correlation between the optimal simulated diffraction template (open red circle overlay) and an experimental diffraction pattern acquired in the 60° tilt dataset. In a nanocrystalline sample, complex diffraction patterns may emerge at specific beam positions due to the possibility of overlapping grains along the beam direction. This is seen by the extra diffraction spots not matching the template in Figure 2a. A multi‐indexing extension of ACOM has been employed to retrieve information from overlapping grains^[^ ^40^ ^]^ In this algorithm, the reflections corresponding to the best‐matching template from the first indexation step are removed and the remaining diffraction spots are re‐indexed, revealing multiple crystal orientations in the same diffraction pattern. This iterative process revealed the orientation of all overlapping grains. Figure S1 (Supporting Information) exemplifies this strategy for the 60° tilt dataset.

The projected images of individual grains were then obtained by using virtual dark field imaging,^[^ ^41^, ^42^ ^]^ as shown in Figure 2b. Here, multiple virtual apertures are positioned around the experimentally obtained diffraction spots according to best‐matched diffraction templates as shown in Figure 2a. Only the diffraction signal within these virtual apertures is integrated to reconstruct the corresponding projected image of a grain (see Figure 2b).^[^ ^17^, ^43^ ^]^ To account for the presence of twins in the sample, a twin orientation‐specific template scheme was used to automatically separate twins from matrix reflections, as illustrated in Figure S2 (Supporting Information). The separation of twin and matrix projection signals is a prerequisite for consistent tomographic shape reconstruction in materials exhibiting this type of orientation relationship^[^ ^44^ ^]^ As an example, an isolated grain containing twin domains was tracked for the entire tilt range as shown in Figure 2c.

Prior to the 3D reconstruction of each grain, intensity normalizations accounting for the decay of the electron probe and for differences in diffraction conditions between different tilt settings were applied. Each projection was first normalized by the diffraction frame total image, calculated by summing the total transmitted and diffracted beam intensities, and was then subsequently renormalized to ensure that the summed virtual dark field intensity of each grain projection remained constant across all tilt angles^[^ ^44^ ^]^ The series of projected images for each grain was first aligned in the IMOD software using a global set of alignment parameters for the sample^[^ ^45^ ^]^ Subsequently, the grains were individually reconstructed in 3D using the Simultaneous Iterative Reconstruction Technique (SIRT) algorithm with 15 iterations combined with a non‐negative minimum constraint to promote physical solutions. The 3D reconstruction of the grain shown in Figure 2c is illustrated in Figure 2d. Each grain was reconstructed independently using the global set of alignment parameters and located into a final common reconstruction volume. Hence, the reconstructed volume of all crystals in the dataset was colored according to their crystallographic orientation along *z*‐axis as shown in Figure 2e (video in Video S1, Supporting Information) for two different view angles.

### 2.3 3D Grain Boundary Mapping

The reconstructed volume (256 × 74 × 74 voxel grid) shown in Figure 2e consists of nano‐sized Ni‐W grains which are separated by general GBs as well as coherent and incoherent twin boundaries. Since the crystallographic orientation of each grain is known, the grain misorientation and GB plane normal can be obtained after the conversion of the voxelated data into a 3D GB mesh. Details on the procedure using a marching cubes algorithm^[^ ^39^ ^]^ are described in the method section. The 3D GB normal orientation plot is thus represented as a surface plot with two adjacent surfaces (see Figure 2f,g). The local GB normal direction is converted to the adjacent crystal reference frames and colored following the crystallographic IPF color code as shown in Figure 2e. A zoomed‐in view of the GB plane normal plot containing the twin boundaries (Figure 2a–d) together with the corresponding grain boundary plane normal distribution (GBND) is shown in Figure 2g. The color of the two twin boundary surfaces suggests an <111> orientation of the surface, respectively, interface normal, corresponding to a coherent Σ3 twin boundary in a fcc crystal. This is confirmed when looking at the GBND shown in the bottom part of Figure 2g. Since this section of the reconstructed volume (highlighted in Figure 2f) is populated with Σ3 annealing twin boundaries with a common {111} twin boundary plane, the GBND shows a preferred distribution of the interface planes of <111>.

### 2.4 Correlative 3D Atom Probe Tomography

**Figure 3:**
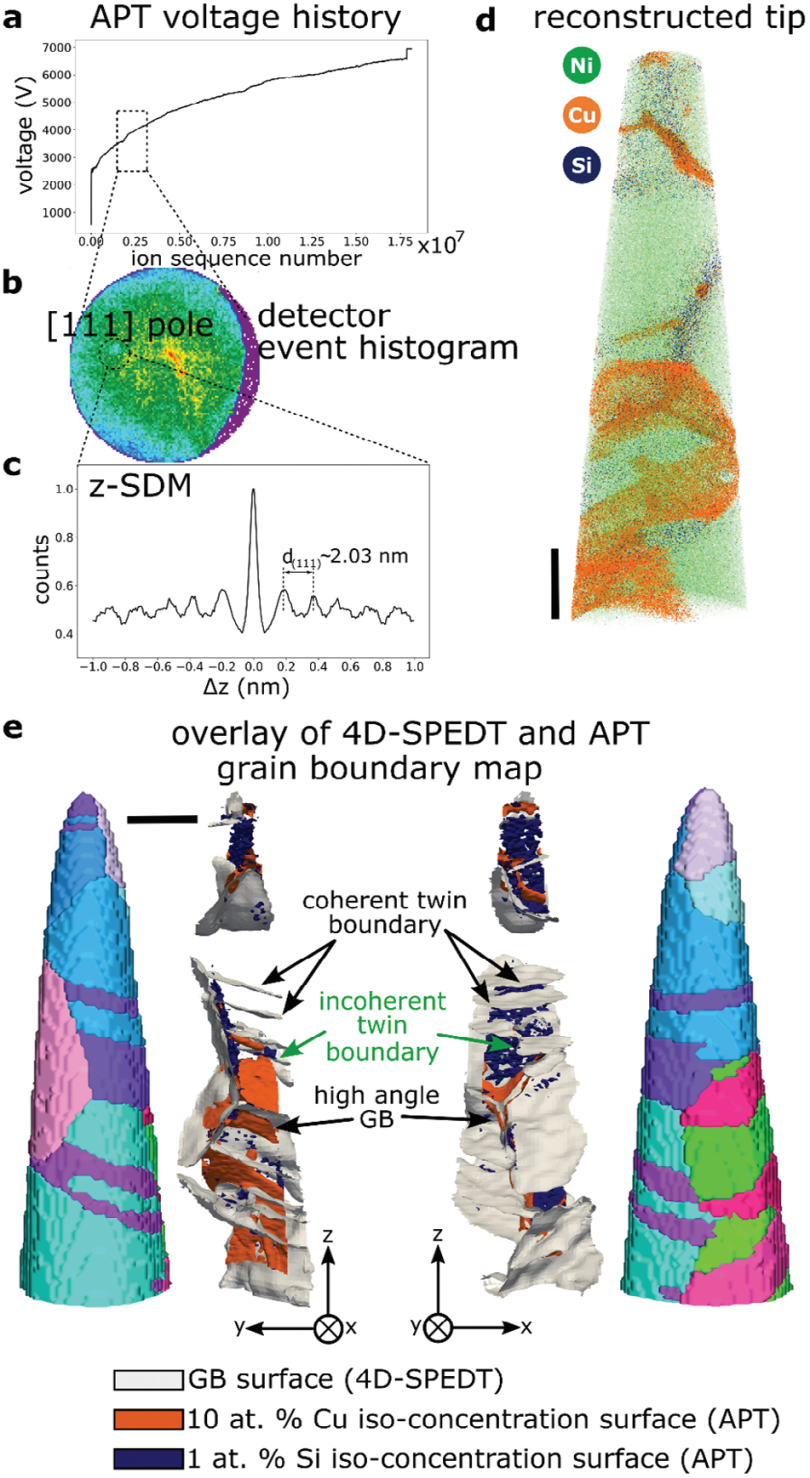
a) The voltage curve for the sample until fracture. The region highlighted by the black rectangle was used to generate the detector event histogram in (b). b) The detector event histogram with multiple ion events revealing a crystallographic pole, here <111>, along the tip axis (*z*‐axis). c) z‐SDM of all ions in the selected sub‐volume enabling to refinement the reconstruction parameters to match the spacing of the lattice planes to {111} fcc of Ni. d) 3D atom map of the reconstructed volume displaying the distribution of Ni, Cu, and Si atoms. e) Overlay of iso‐concentration surfaces of Cu (10 at.%, orange) and Si (1 at.%, blue) obtained by APT on the GB surface network (gray surface) reconstructed from 4D‐SPEDT (shown in Figure 2e) for two different view angles. The corresponding 3D crystal orientation map is shown for comparison. Scale bars are 50 nm.

The same sample was subsequently field‐evaporated (electric and laser pulse) in the analysis chamber of an APT instrument, as detailed in the method section. The top atomic layers of the needle‐shaped specimen are evaporated by short laser pulses. The evaporated ions are accelerated toward a position‐sensitive detector by applying an electric field just below the field evaporation threshold (see **Figure** **3**a,b). By measuring the time‐of‐flight of the ions and their mass‐to‐charge ratio, it is possible to back‐project the 3D positions of the ions into the volume of the original sample (see Figure 3d). This process is repeated until a sufficiently large number of ions, typically >10^6^ have been detected. To obtain an accurate 3D reconstruction, the reconstruction parameters need to be carefully calibrated. Gault et al. developed an approach to calibrate the parameters on lattice planes when multiple crystallographic poles are imaged within a single grain in the APT data^[^ ^46^ ^]^ However, the sample investigated in this study is a complex alloyed material system with a nanograined structure, and obtaining crystallographic poles throughout the entire volume is challenging^[^ ^47^ ^]^ When selecting a specific ion evaporation sequence from the voltage history curve (marked by black dashed rectangular box in Figure 3a), it is possible to select a specific grain or crystal from the 3D reconstructed volume shown in Figure 3d. The corresponding detector event histogram shown in Figure 3b shows the presence of a crystallographic pole, indicating that the crystal here was oriented close to {111} pointing toward the apex of the needle‐shaped specimen. Choosing a cylindrical region (≈2 nm diameter and ≈10 nm depth) around the pole on the detector event histogram allows to selections from the 3D reconstruction with {111} planes being perpendicular to the evaporation direction. This enables to resolution the {111} lattice planes in the z‐spatial distribution map (SDM) as shown in Figure 3c. We utilize the 4D‐SPED data to confirm the orientation of the {111} lattice planes for this particular crystal. Hence, the optimized reconstruction parameters are applied to obtain the entire 3D atom map as shown in Figure 3d (video in Video S2, Supporting Information). It becomes apparent that Cu and Si atoms are predominantly present at the GBs, which would otherwise be difficult to identify in APT, as illustrated in Figure 3d.

Since we now have the 3D crystal orientation and the corresponding 3D elemental distribution available from the same sample, it is possible to explore the 3D character of GB segregation. To isolate GB segregation in the APT data, iso‐concentration surfaces of ≈10 at% Cu (orange) and ≈1 at% Si (blue), determined by manual inspection, are extracted and overlaid on the reconstructed GB network (gray surface) from the 4D‐SPEDT data as shown in Figure 3e. The entire grain reconstruction (in IPF color coding) from 4D‐SPEDT is also shown alongside the same orientation for comparison in Figure 3e. This illustrates that overall, there is a good correlation between the 4D‐SPEDT and APT datasets, especially considering that the APT reconstruction was refined using crystallographic information, but no further processing was applied. On the bottom side of the reconstruction away from the tip apex, one can observe a slightly larger deviation between the GB surface reconstruction and the iso‐concentration surfaces, which is related to limitations in the APT reconstruction.

### 2.5 Linking Grain Boundary Character and Composition in 3D

**Figure 4:**
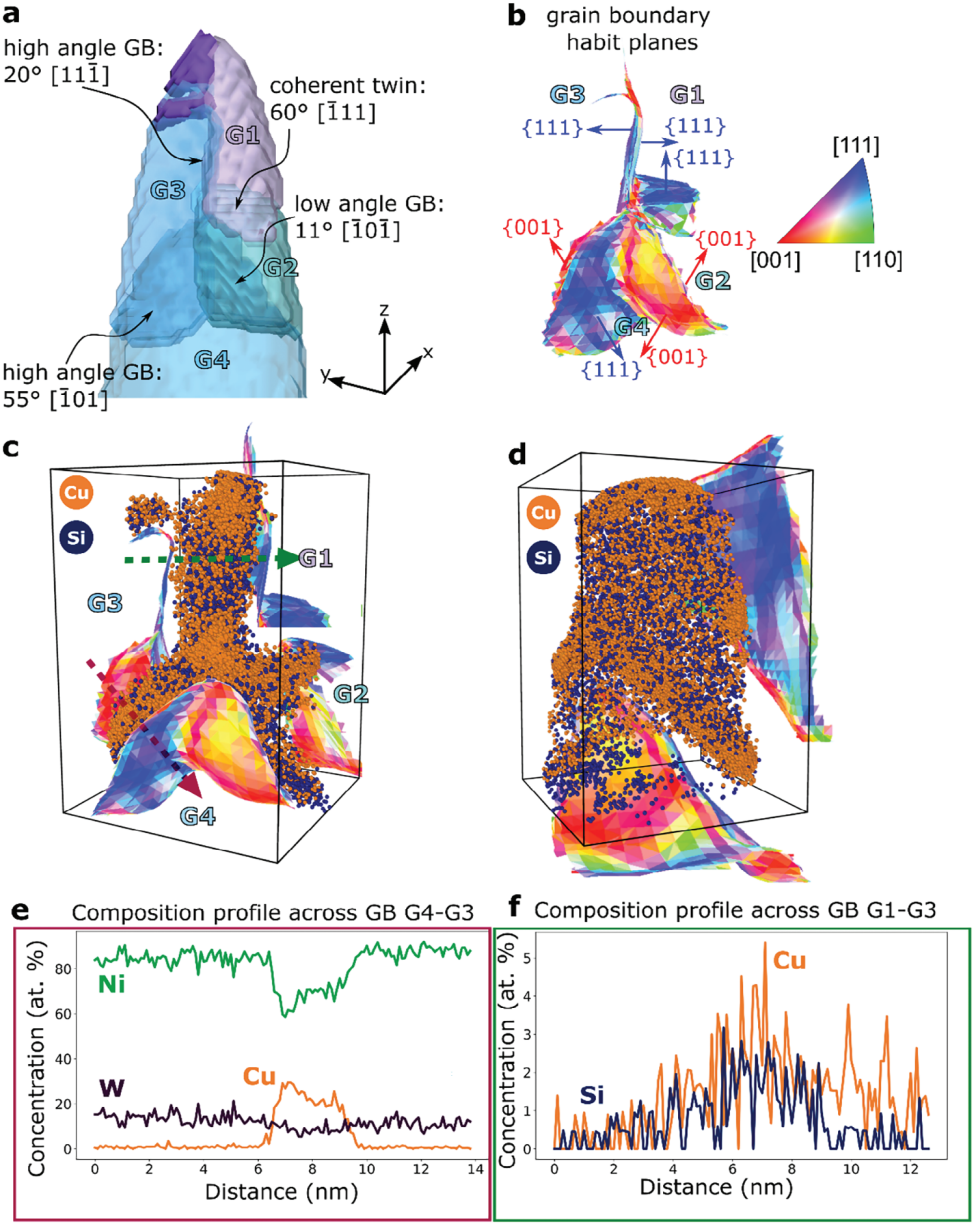
a) 3D crystal orientation of the tip of the needle‐shaped sample with indicated GB types, their corresponding misorientation angle, and global GB habit plane. b) GB habit plane map colored according to the IPF color code. The GB habit plane map consists of two closely spaced surfaces corresponding to the GB plane normal of the adjacent crystals. c) Exploded view of the GB habit plane map showing the two surfaces superimposed on the corresponding 3D atom map of Cu and Si obtained by APT. d) The same explosion view as in (c) in a different view angle showing the GB between G1 and G3 plane on. e) Concentration profile extracted across the high angle GB G3‐G4 showing strong Cu enrichment. The Si profile is not shown here since it does not show any significant sign of segregation. f) Concentration profile extracted across the high angle GB G1‐G3 with a slight indication of Cu and Si segregation.

To demonstrate the direct 3D linking of the GB character with solute segregation, we focus on two selected parts of the reconstruction shown in Figure 3e. The grain reconstruction, atom maps, GB character, and composition of the region close to the apex of the needle‐shaped sample are shown in **Figure** **4**. In the analyzed volume, a strong segregation tendency of Cu along general highangle GBs is observed, whereas Si atoms preferentially decorate low angle grain boundaries GBs and a high angle GB with a misorientation of 20°. We mostly find low energy Σ3 boundaries in the volume analyzed here and the other interfaces are non‐CSL type GBs.

A coherent Σ3 60° {111} twin boundary is observed between grains G1 and G2, which does not show any sign of solute segregation as expected^[^ ^48^ ^]^ The low angle GB surrounded by grains G2 and G4 with a misorientation angle of 11° exhibits similar GB plane normals of the abutting grains of {100}_G2_/{100}_G4_, which is primarily decorated by Si atoms and no Cu segregation is found (see Figure S3a, Supporting Information). Regularly spaced peaks of Si are observed in the Si atom density map of this low angle GB, which suggests that Si atoms are segregating to the array of dislocations in the interface as shown in Figure S3b (Supporting Information). Strong Cu segregation of more than 30 at.% in peak concentration is observed at a general high angle GB with a misorientation angle of ≈55° in between grains G4 and G3 (GB plane normals {111}_G4_/{100}_G3_) as shown in Figure 4e. Interestingly, W seems to be slightly depleted at the GB, and the Si concentration profile (see Figure S4, Supporting Information) does not show any significant sign of segregation. Such strong Cu segregation to a general high angle GB having nearly low‐indexed GB planes deviates from previous findings, where typically low segregation values are found for such types of interfaces.^[^ ^49^, ^50^ ^]^ The high angle GB separating grains G1 and G3 has a global GB plane normal close to {111}_G1_/{111}_G3_ and a misorientation angle of ≈20°. This GB is decorated with both Cu and Si atoms as can be seen in the concentration profile shown in Figure 4f, but with a much lower content of the solutes compared to the GB between grains G3 and G4. The lower content of Cu at the 20° compared to the 55° GB could be related to several factors including the density of secondary GB dislocations, the GB habit plane normals and local GB parameters.

**Figure 5:**
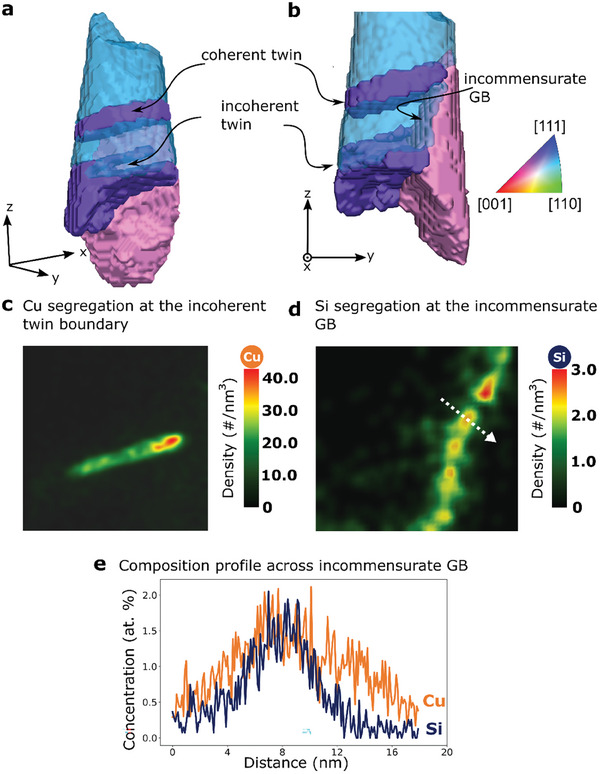
a) A cropped region from 3D reconstructed grains highlights the front (translucent) and back (solid) grains colored according to the IPFcolor code pointing along *z*‐axis. b) GBs of interest (coherent and incoherent twin boundaries, and incommensurate GB) are marked. c) and d) shows Cu atoms and Si atoms density map respectively, on a 2D plane perpendicular to its iso‐concentration surface placed such that the decoration of Cu and Si atoms can be visualized. In (d), a discontinuous enrichment up to 3 atoms nm^−3^ of Si can be visualized. e) 1D compositional profile measured across the incommensurate GbB in the direction of white arrow in (d) showing the maximum enrichment of Cu and Si at this boundary.

**Figure** **5**a–e illustrates the 3D structure‐composition correlation at a stepped twin boundary. The coherent twin boundary between the purple and blue grains shows no segregation, whereas a nanometer scale incoherent twin boundary segment is strongly enriched in Cu along its entire length (see Figure 5c). A GB between the purple and pink grains in Figure 5b is identified as an incommensurate (singular) boundary, a special asymmetric GB with 45° misorientation and GB habit planes of (001)/(011), as discussed by Lejček et al.^[^ ^51^ ^]^ The special nature of this high angle GB seems to limit the segregation of both Cu (≈1.5 at.%) and Si (≈1.5 at.%) (see Figure 5e). A discrete modulated segregation pattern of Si atoms is observed (see Figure 5d), which can indicate a nanometer scale faceting of the boundary.

By correlating 3D crystal orientation mapping and APT on the same sample, we could elucidate the nature of the local GB structure and composition in a nanocrystalline material. The rich multidimensional data provides novel insights into the segregation behavior with nanometer resolution in 3D and provides access to a large fraction of GB‐types, but is also capable to reveal often overlooked features, such as a nanometer‐sized incoherent GB segment. This experimental evidence lays the foundation to build atomistic and thermodynamic models to understand the underlying segregation mechanisms. For example, W segregation was only observed near triple junctions and to GBs in their close proximity. It seems that Cu displaces W during the annealing treatment from most of the GBs and also impacts the extent of Si segregation. Furthermore, the GB character‐dependent Cu segregation here deviates from the predicted GB segregation energy spectra of Cu at GBs in Ni polycrystals using the embedded atom potential method.^[^ ^52^, ^53^ ^]^ Such discrepancies between atomistic simulations and experimental observations require a more holistic characterization of GBs and their related segregation behavior to ultimately build better models to predict interfacial properties. Our direct 3D correlation of GB structure and compositions opens this field and provides pathways to study multi‐elemental segregation at complex 3D GB networks.

## 3 Discussion

In summary, a framework to probe the 3D nature of the structural parameters and composition of GBs is presented. We combine 4D‐SPEDT (3D crystal orientation mapping) with APT (3D compositional information) to obtain a correlation of the macroscopic degrees of freedoms of interfaces along with their compositional fingerprint. In particular, we demonstrate a methodology to determine the 3D GB habit plane network on a quantitative basis, which is the foundation for understanding how the GB character affects elemental segregation, which ultimately determines the properties of a material. It has been shown that GB segregation has the potential to engineer material properties^[^ ^31^ ^]^ In metallic alloys, GB segregation can be used to impede grain coarsening and thus maintain a higher strength of the material or to improve interface damage resistance^[^ ^31^ ^]^ GB segregation in nanostructured catalyst materials stabilizes the nanocrystalline grain structure making them more durable under electrocatalytic conditions^[^ ^35^ ^]^ Furthermore, localized oxidation at GBs and related ingress of oxygen is important to be considered in the activity and stability evaluation of electrocatalytic materials.^[^ ^31^, ^34^ ^]^ A structurally and chemically complex nanocrystalline material is chosen to show the strength of the 3D correlative framework. Besides the local GB character, salient features such as incoherent twin boundary segments can be revealed both structurally and chemically by unleashing the strength of each of the techniques. The 3D structure‐composition data opens the door to studying complex interfacial networks in polycrystalline nanomaterials at nanometer resolution and provides novel insights into complex materials required for making accurate predictions of interfacial properties and with this material behavior. In many cases, crystallographic information in nanostructured materials can hardly be discerned in APT due to the complex nature of the field evaporation process. Currently, the spatial resolution of our method on the electron microscopy side is limited by the attainable nanoprobe size and the number of projections used in the reconstruction, which is limited by the speed of the pixelated detector. However, advancements in electron optics and detector technologies for 4D‐STEM will allow for further reduce the electron probe size and data acquisition times to increase resolution and eventually probe GB segregation phenomena during annealing treatments, electrochemical testing or to determine localized strain build‐up at the interfaces as well as possibly related elastic properties in 3D.^[^ ^31^, ^34^, ^35^, ^54^ ^]^ Although a needle‐shaped sample geometry is required for 3D crystal orientation mapping and correlated APT measurements, it can be envisioned in the future to use the 3D crystallographic data to locally refine the APT reconstruction on a 3D voxel basis by data fusion techniques to ultimately enhance spatial resolution. Our results show the great potential of this new technique in the field of correlative microscopy and its application to GB engineering to facilitate the development of advanced material systems.

## 4 Experimental Section

### Materials

An electrodeposited nanocrystalline (nc) Ni‐14 at.% W alloy on a Cu substrate was received from Xtalic Corporation, USA. The electrodeposition route produces a randomly oriented nano‐grained material^[^ ^55^ ^]^ The sample was annealed at 600 °C for 6 h to crystallize the initially amorphous material in the as‐deposited state and to initiate diffusion of Cu and Si along the GBs of Ni–W nc‐alloy.

### Correlative Sample Preparation Using Focused Ion Beam (FIB)

Needle‐shaped sample preparation for the correlative experiment was carried out using a dual‐beam focused ion beam (FIB) instrument (Thermo Fisher Scientific Helios Nanolab 600i). The sample was lifted out and mounted onto the APT‐compatible cylindrical Cu post of the Fischione on‐axis rotation tomography holder. The apex of the Cu post was pre‐sharpened using FIB to mount the sample. Subsequently, the sample was thinned down to a needle shape at 30 kV accelerating voltage using a Ga ion beam with a current range from 2.5 to 0.8 nA. A final cleaning procedure at 2 kV and 16 pA current was carried out to remove severely damaged regions during thinning at high energy (30 kV).

### 4D‐Scanning Precession Electron Diffraction Tomography

The 4D‐SPED was performed in a JEOL JEM‐2200FS microscope operated at an accelerating voltage of 200 kV using a 10 µm condenser (CL1) aperture and a calibrated camera length of 400 mm (calibrated using ASTAR software package) using 30 eV energy filter. The probe size was estimated to be ≈2.5–3 nm after a precession angle of 1° at 100 Hz was applied by the Digistar hardware unit (NanoMEGAS SPRL). The precessed nano‐probe beam is scanned across the needle‐shaped sample with a step size of 2.4 nm and nanobeam diffraction patterns are acquired at each probe position with a 4k × 4k CMOS detector (TemCam‐XF416‐TVIPS) hardware‐binned to 512 × 512 pixels^2^ The diffraction patterns were further binned numerically to 256×256 pixels^2^ using the tvipsconverter software for further processing^[^ ^56^ ^]^ To speed up the acquisition time, the needle‐shaped sample was aligned along the fast scan direction, and a frame comprising 100 × 276 pixels^2^ was used to scan the sample. A pixel dwell time of 41 ms was used to capture each nanobeam diffraction pattern, resulting in ∼4 rotations of the precessed incident beam per frame, which significantly reduces dynamical diffraction effects. A 4D‐SPED scan was recorded over a sample tilt range from −80° to +80° with a tilt step increment of 10°, for a series of 17 projections in total. A Fischione Instrument Model 2050 On‐Axis Rotation Tomography Holder was used to acquire the tilt series. This results in a complex 3D 4D‐SPED dataset consisting of 469200 precessed, nanobeam electron diffraction patterns in total.

### Atom Probe Tomography

After the 4D‐SPEDT acquisition, the sample was showered by low‐kV Ar ions in a PECS Model 682 system by Gatan at 2 kV and 32 µA current to remove hydrocarbon layers formed during the TEM measurement before loading the sample into the APT analysis chamber^[^ ^57^ ^]^ The APT measurement was conducted using an atom probe 5000XR instrument (LEAP, Cameca Instruments). Laser pulsing mode was applied at a pulse repetition rate of 200 kHz and a pulse energy of 40 pJ. The sample's base temperature was kept at 70 K and a target detection rate of 1% was set. Data analysis was performed using the APsuits software package.

### Data Processing and Analysis

The 4D‐SPED data was processed using the ASTAR software package and in‐house developed Python codes mainly relying on the NumPy, SciPy, and orix software packages.^[^ ^58^, ^59^, ^60^ ^]^ The azimuthal rotation of the diffraction pattern with respect to the rotation axis of the sample holder was calibrated using the procedure described in the paper^[^ ^17^ ^]^ The computed rotation vector (axis and angle) couples the tilt series. A multi‐indexing approach was employed to retrieve through‐thickness information and to treat overlapping grains^[^ ^40^ ^]^ Orientation‐specific virtual apertures were selected for generating the projected virtual dark field images of individual grains throughout the tilt series. A coarse alignment of the projection images in the tilt series is performed manually in the Tomviz software^[^ ^61^ ^]^ Fiducial alignment as implemented in the IMOD software was employed for fine alignment before tomographic reconstruction using the “Simultaneous Iterative Reconstruction Technique” (SIRT) algorithm. Tomviz and Paraview were used for 3D visualization and rendering^[^ ^62^ ^]^ In order to perform correlative data analysis, the axes were defined according to APT convention.

## Conflict of Interest

The authors declare no conflict of interest

## Supporting information



Supporting Information

Supplemental Video 1

Supplemental Video 2

## Data Availability

The data that support the findings of this study are available from the corresponding author upon reasonable request.
