# Supplementary material for: Correlating Grain Boundary Character and Composition in 3‐Dimensions Using 4D‐Scanning Precession Electron Diffraction and Atom Probe Tomography
Source: Small Methods. 2025 Feb 28;9(5):2401650. doi: 10.1002/smtd.202401650 (PMC12103235; doi:10.1002/smtd.202401650)
Supplement: Supplementary file 1 — Supporting Information [file SMTD-9-2401650-s001.docx]

**Supplementary Information**

**Correlating grain boundary character and composition in 3-dimensions using 4D-scanning precession electron diffraction and atom probe tomography**

Saurabh M. Das^1*+^, Patrick Harrison^2+^, Srikakulapu Kiranbabu^1^, Xuyang Zhou^1^, Wolfgang Ludwig^3,4*^, Edgar F. Rauch^2^, Michael Herbig^1^, Christian H. Liebscher^1,5,6*^

^1^ Max-Planck-Institut for Sustainable Materials (Max-Planck-Institut für Eisenforschung), Max-Planck-Straβe 1, 40237 Düsseldorf, Germany

^2^SIMAP Laboratory, CNRS-Grenoble INP, BP 46 101 rue de la Physique, 38402 Saint Martin d'Hères, France

^3^ESRF–The European Synchrotron, 71 Av. des Martyrs, 38000 Grenoble, France

^4^MATEIS, INSA Lyon, UMR 5510 CNRS, 25 av Jean Capelle, 69621 Villeurbanne, France

^5^Research Center Future Energy Materials and Systems, Ruhr Univeristy Bochum, Universitätsstr. 150, 44801 Bochum, Germany

^6^Faculty of Physics and Astronomy, Ruhr Univeristy Bochum, Universitätsstr. 150, 44801 Bochum, Germany

*Corresponding authors,

email: 100rabh2992@gmail.com (Saurabh Mohan Das), wolfgang.ludwig@esrf.fr (Wolfgang Ludwig), christian.liebscher@rub.de (Christian H. Liebscher)

^+^These authors contributed to the work equally.


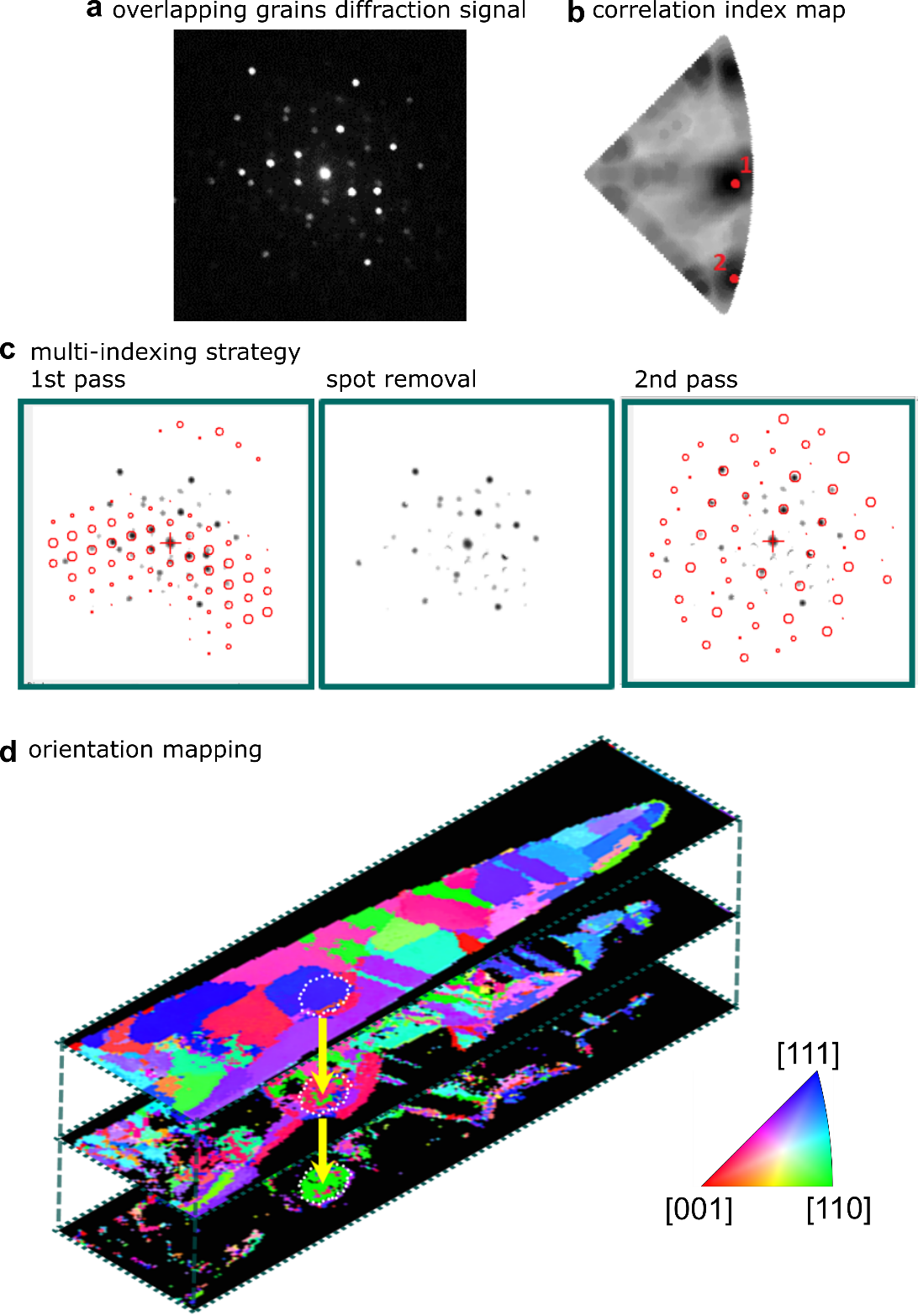


**Figure S1:** Multi-indexing strategy reveals the hidden grain along the beam direction. (a) A diffraction pattern from the 60° tilt dataset containing signal from overlapping grains. (b) Correlation index map showing the two possible solutions marked as 1 and 2. (c) ACOM best-matched template overlay (left), the new diffraction pattern after removing the diffraction signal corresponding to the best-matched template (middle), and (right) the 2^nd^ best-matched template corresponds to the non-dominant grain. (d) Orientation mapping of a 60° tilt data set taken as an example of multi-indexing strategy reveals a grain (highlighted with a dotted white circle) with orientation close to (110) that only is indexed in the 2^nd^ and 3^rd^ indexing passes.


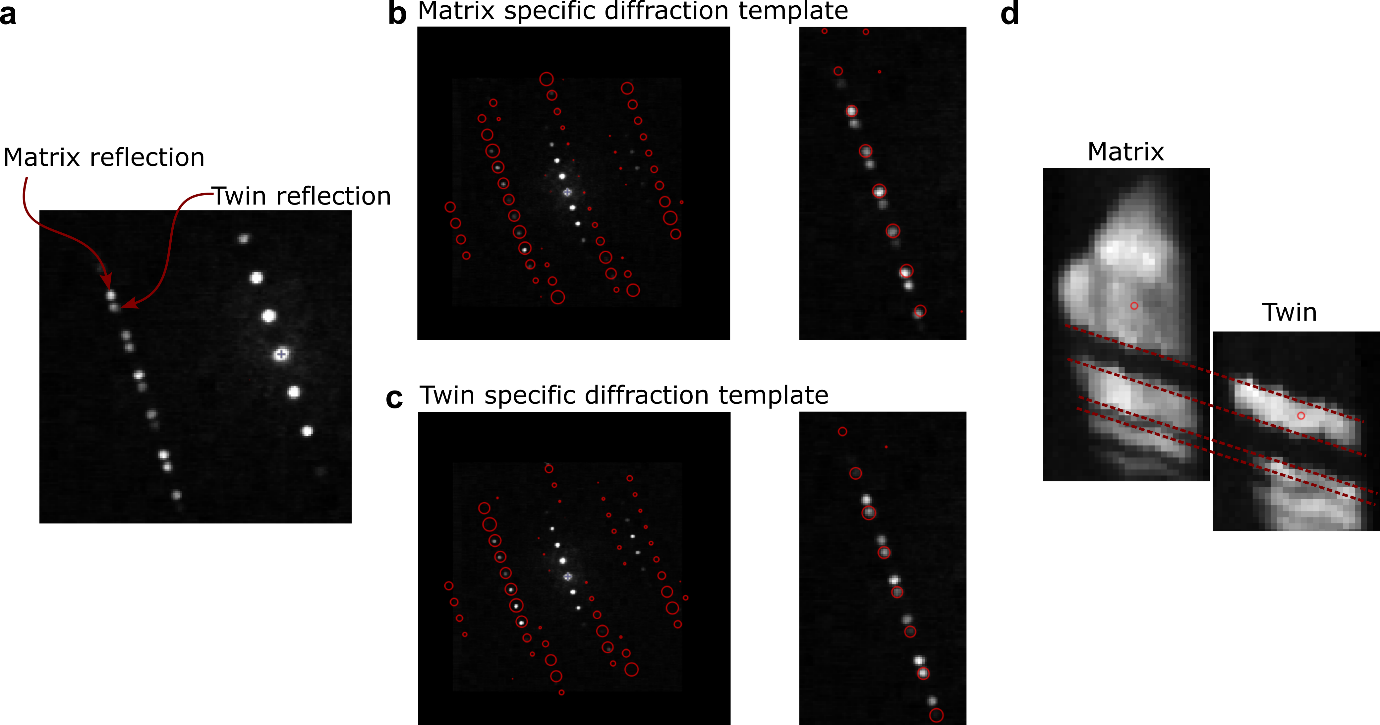


**Figure S2:** Twin reconstruction strategy. (a) Cropped region of diffraction pattern showing the matrix and twin reflections. (b) Matrix-specific diffraction template, and (c) twin-specific diffraction templates are used to separate the matrix and twin reflections, and (d) subsequently these specific templates are used as virtual apertures to generate the matrix and twin projection images using Virtual Dark Field imaging algorithm^1^.


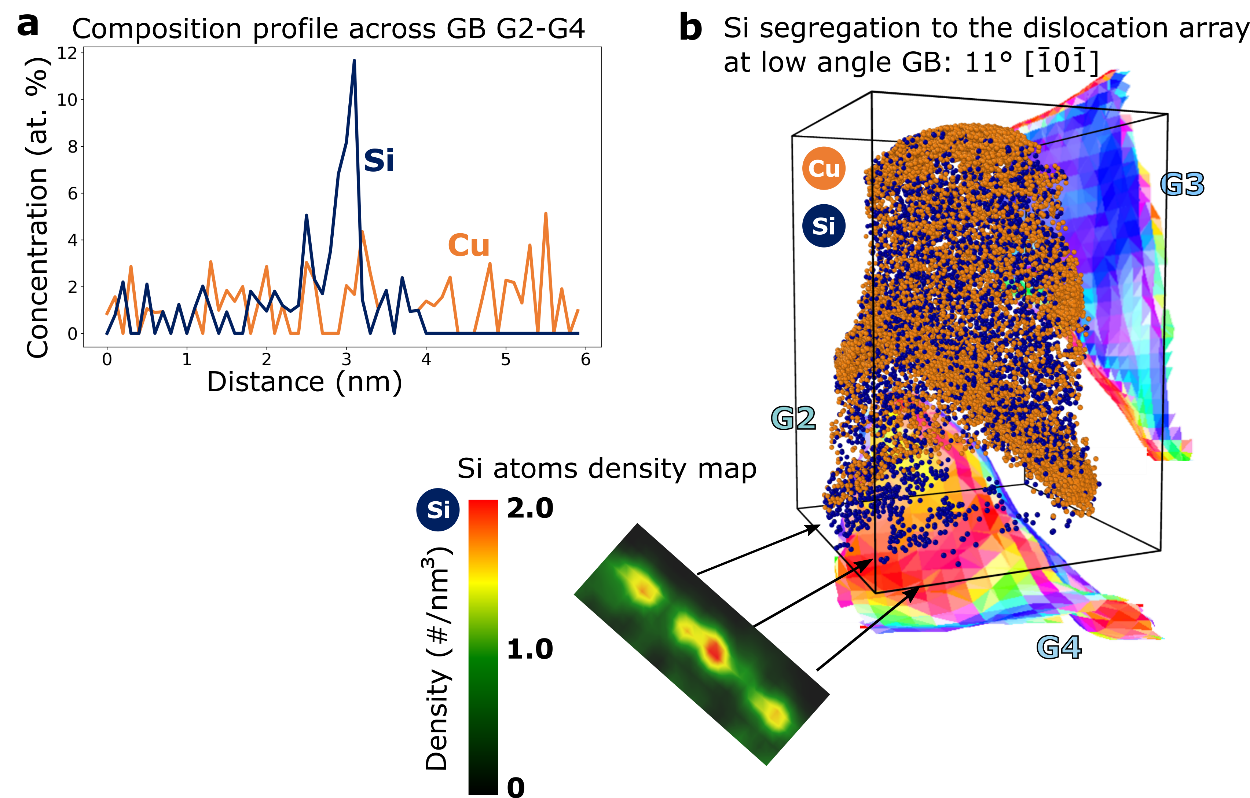


**Figure S3:** Si atoms are segregating to the array of dislocations at G2-G4 low angle GB. (a) Si and Cu concentration profiles across G2-G4 GB show only Si enrichment at the dislocation. (b) Si atoms density map on a 2D plane perpendicular to its iso-concentration surface across G2-G4 GB, placed such that the decoration of dislocation core is visualized.

**
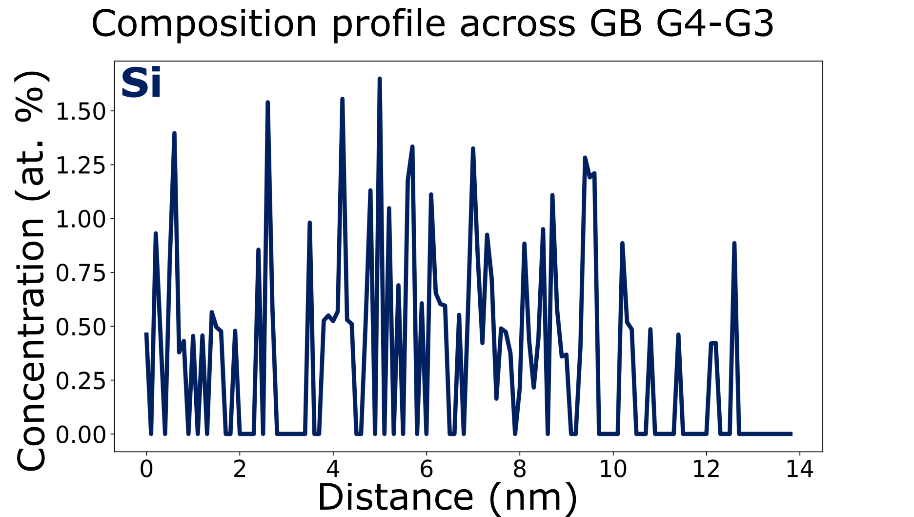
**

**Figure S4:** Si concentration profile does not show any significant sign of segregation across G4-G3 high angle GB.

**Reference:**

1.P. Harrison, S. M. Das, W. Goncalves, A. da Silva, X. Chen, N. Viganò, C. H. Liebscher, W. Ludwig, X. Zhou, E. F. Rauch, Ultramicroscopy 2024, 267, 114038.
